# Exploring relational and moral features in medical students

**DOI:** 10.5116/ijme.606a.f16c

**Published:** 2021-04-29

**Authors:** Joana Moreira Pereira, Luís Madeira, Maria Luisa Figueira

**Affiliations:** 1 Department of Psychiatry, Centro Hospitalar Lisboa Norte, Portugal; 2 Department of Psychiatry, Centro Hospitalar Psiquiátrico de Lisboa, Portugal

**Keywords:** Empathy, moral competences, callous traits, temperament, medical education

## Abstract

**Objectives:**

We aimed to explore empathy, moral competencies, callous traits, and temperament in a sample of medical students. Furthermore, we aimed to investigate differences in our variables across the 1st and 5th years of medical education and possible correlations between them.

**Methods:**

This was a cross-sectional study with 138 medical students. We resorted to self-reported instruments that were given at the end of classes: Barrett-Lennard Relational Inventory, Temperament Evaluation of Memphis, Pisa and San Diego Auto-questionnaire, Inventory of Callous-Unemotional Traits, and Moral Competence Test. For the statistical analysis, we resorted to descriptive and inferential statistics, using non-parametric tests when data didn't follow a normal distribution.

**Results:**

We found no statistical difference between empathy scores in 1st (N=104, Mean=41.42, SD=22.48) and 5th year students (N=34, Mean=37.35, SD=23.35), t_(136)_=0.908, p=0.366. Callous traits were negatively correlated with empathy (r_(136)_=-0.444, p=0.000) and no correlation between moral competences and empathy (r_(96)_=0.029, p=0.779) was observed. We found a negative correlation between empathy and cyclothymic, anxious and irritable temperaments (r_(136)_=-0.334, p=0.000, r_(136)_=-0.281, p=0.001, r_(136)_=-0.400, p=0.000).

**Conclusions:**

Our scores corroborate previous evidence that medical students are empathic, have good moral standards and low callous traits. We saw no differences in empathy scores between the two years and future studies could explore the particulars of medical curriculums impacting this variable. In our study, empathy was negatively correlated with callous traits and linked with specific temperaments. Considering these variables at admission to medical school as well as preserving and improving them in medical education might offer better standards of care.

## Introduction

Relational and moral competences are key features in medical practice, as they shape the way the encounter with patients unfolds and our decision making. The clinical encounter should provide a safe and warm atmosphere where patients fully disclose their symptoms and explore fears, reservations, and concerns, embarking on a reliable and effective therapeutic project. Shared decision making in medicine is always a dialogical process and tailor-made interventions must respect both patients' autonomy and beneficence in accordance to the leges artis. Despite the relevance of relational and moral competences on clinical practice these aren't routinely measured when choosing candidates for medical school and they are also dismissed in most medical education curriculums.

First, empathy is key to relational competences in medicine, allowing the patient to experience presence, warmth, and understanding.^1^ Introduced in medicine in 1963 by William Osler, empathy refers to a special form of sharing patients' experience (different from contamination or neutralization of emotions) in which it is possible to "see inside" and characterize the inner life of patients. It requires a cognitively challenging^2^ embodied recognition of another's emotional state and the identification of behavioral correlates, personality dimensions, and emotions.^3^ For a tentative operational definition four levels were suggested: emotive (the ability to subjectively experience and share the other's psychological state and feelings), moral (an altruistic internal force), cognitive (the intellectual ability to identify and perceive the other's perspective in an objective way), and behavioral (communicative response in order to show insight into the other's perspective).^4^ The development of empathy across medical pre-graduate curriculums was explored in order to understand possible differences across students and which factors reduced and promoted it. Students that start their studies with lower scores appear to get a greater reduction of empathy^5^ and is evidence towards empathy fading from the first year of study.^6^^,^^7^ It is postulated that the rigor of the theoretical curriculum, which models the academic life, leads students to dismiss communicative and empathic skills.^8^ Other assorted explanations for the decline of empathy include 1) "de-idealization" of medicine (expectations vs reality), 2) defense mechanisms to distance themselves from illness or death^9^ and 3) the risk of litigation, economic and time constraints.^10^ This last factor is part of today's health systems requirements (that favor productivity over quality and fast interventions) and leads to a guarded, impersonal, and distant communication style.^11^ Interestingly, previous studies with Portuguese samples seem to show a conservation or even increase in empathy.^12^

Temperament also has an important role on relational competences as it shapes the way individuals interact with one another. For example, irritable temperament is associated with lower levels of empathy, anger, and violence whereas depressive temperament is characterized by perseverance and reliability.^13^ Temperament has been considered since Kraepelin and the present-day definition by Akiskal as "a hereditary core of the personality that determines reactivity, mood and energy of a given individual and that it is relatively unchangeable throughout life".^14^ It possesses both positive and negative aspects, impacting the development of personality and quality of life, being linked to some personality traits.^15^

In our study, morality construct is addressed both positively as competences in moral judgements and negatively by studying callous and unemotional traits (CUT). Moral judgment as a broad category can be clarified by the analysis of paradigmatic cases. The Moral Competence Test (MCT) is a good tool to identify moral decision making. CUT are part of the affective dimension of psychopathy, including the narcissistic traits and manipulative, impulsive, and irresponsible behavior. They commonly refer to persons who lack empathy and/or guilt. More specifically, people with higher CUT "are more likely to show deficits in their processing of negative emotional stimuli, low levels of fearful inhibitions and anxiety and decreased sensitivity to punishment cues, especially when a reward-oriented response set is primed".^16^^,^^17^ Morality is paramount to medicine considering the amount of ethical decisions that are involved in everyday practice.

Our study aimed at a comprehensive larger analysis of these competences in a sample of medical students including their possible inter-relations. Our first aim was to clarify our sample empathy, morality, callous traits, and temperament scores and to identify possible differences between 1st and 5th year students. Our second aim was to identify whether empathy correlated with morality (positively) and callosity (negatively). Our last aim was to identify the correlation between specific temperaments and our measured variables (BLR, ICU and MCT)

## Methods

### Study design and participants

This is a cross-sectional study with a sample of 138 students from Lisbon University Medical School. Although we aimed to include all students from the 1st and 5th year (no exclusion criteria were defined), we got a 42 % response rate. All participants were recruited between 1 August 2018 and 1 February 2019 and signed a written voluntary informed-consent form prior to participation. The sample comprised 34 (24.6%) 5th-year students and 104 (75.4%) 1st-year students. Voluntary participation was guaranteed, and confidentiality was assured by random code allocation of each student participation. The study was approved by Ethics Committee of Centro Académico de Medicina de Lisboa (Centro Hospitalar Lisboa Norte and Faculdade de Medicina da Universidade de Lisboa).

The subjects were between 18 and 30 years old, with a mean age of 20.58 (SD: ±0.325), of which 89 (65.2%) were female. Regarding the family history of psychiatric disorder, 10 (7.2%) students reported a family history of mood disorder, 7(5.1%) reported anxiety disorders, 2 (1.4%) reported schizophrenia and 1(0.7%) reported alcohol use disorder. Regarding the current prescription of psychotropic drugs, 4(2.5%) students reported taking antidepressants, 2 (1.4%) reported taking antidepressants and benzodiazepines, 1(0.7%) reported taking benzodiazepines, and 2 (1.4%) reported taking mood stabilizers. Research wise we considered both segregated and combined population of our 1st and 5th-year students – which showed no differences on baseline demographics with the exception of gender as 1st-year students were mainly females. Table 1 presents the full sociodemographic features of our sample.

### Data collection

We administered 4 paper-format self-reported scales in the context of a classroom allowing any question during the process to be clarified. The scales included Temperament Evaluation of Memphis, Pisa and San Diego Auto-questionnaire (TEMPS-A) to assess temperament, Inventory of Callous-Unemotional Traits (ICU-T) to assess callous traits, Barrett Lennard Relational Inventory (BLRI) to assess empathy, and the Moral Competence Test (MCT-T) to assess morality competence. Validity and Reliability are presented in each of the scales.

**Table 1:** Sociodemographic and clinical characterization of the sample

Variable		Total Sample (N=138) Mean ± SD n (%)	1^st^ Year (N=104) Mean ± SD n (%)	5^th^ Year (N=34) Mean ± SD n (%)	F or Fisher's	p
Age		20.58±3.251	19.88±3.454	22.71±0.719		
Country						
	Portugal	126 (91.3)	95(91.3)	31(91.2)	1.000	0.607
	Other	12(8.7)	9(8.7)	3(8.8)		
Gender						
	Male	48(34.8)	28(26.9)	20(58.5)	1.000	0.002*
	Female	90(65.2)	76(73.1)	14(41.2)		
Ethnicity						
	Caucasian	131(94.9)	99(95.2)	32(94.1)	1.000	0.550
	Other	7(5.1)	5(4.8)	2(5.9)		

N= number of participants or Mean ± Standard deviation. Percentages under parenthesis. p-values refer to Fisher's Exact Test between 1st and 5th year medical students for categorical variables. *Significant difference (2-tailed p<0.05)

### Barrett Lennard Relational Inventory (BLRI)

BLRI is a self-report questionnaire, with a six-point bipolar rating scale ranging from -3 ('NO, I strongly feel that it is not true') to +3 ('YES, I strongly feel that it is true') that comprises four subscales: 'Empathic Understanding', 'Level of Regard', 'Unconditionality', and 'Congruence'. Since the internal consistency of the 40-item Version has not been determined, we used the 64-item Version which showed reliabilities of .91 for Level of Regard, .88 for Congruence, .84 for Empathy, .74 for Unconditionality and .91 for the total score.^18^ The total score is the sum of the 64-item answers, being that eight questions per subscale are negative items (scored by multiplying "-1"). The scale was validated to the Portuguese population by Marques-Teixeira and colleagues.^19^

### Temperament Evaluation of Memphis, Pisa and San Diego Auto-questionnaire (TEMPS-A)

TEMPS-A is a 110-item instrument that measures affective temperament traits represented along five dimensions: depressive (questions 1 to 21), cyclothymic (22 to 42), hyperthymic (43 to 63), irritable (64 to 84) and anxious (85 to 110)14. It measures the severity of the traits of the temperament ranging from 0 to 1 and gives the ability of group comparison. However, it does not answer to the question about a person's temperament type, as there is no standard for "normal" temperament. The total score for each subscale is given, dividing the sum of points obtained by the number of questions contained therein.^14^ TEMPS-A has been translated into 32 languages and has demonstrated high reliability and internal consistency^20^^,^^21^ ("Coefficients a for internal consistency were 0.91 (cyclothymic temperament subscale), 0.81 (depressive temperament subscale), 0.77 (irritable temperament subscale), 0.76 (hyperthymic temperament subscale), and 0.67 (anxious temperament subscale)"^22^). The scale was validated to the Portuguese population by Figueira and colleagues.^22^

### Inventory of Callous-Unemotional Traits (ICU-T)

There are five versions of the ICU-T scale:  Youth Self-Report, Parent Report, Teacher Report, Parent Report (Preschool Version), and Teacher Report (Preschool Version). In our study, we used the ICU-T youth version – a 24-item questionnaire scored on an ordinal 4-point Likert scale (from "Does not apply at all" to "Applies very well") in which scores are divided into 3 dimensions: callousness, uncaring and unemotional.^23^ ICU-T provides a comprehensive assessment of callous and unemotional traits, which are relevant for identifying a distinct subgroup group of antisocial and aggressive youth. We used the ICU-Youth Version, translated to Portuguese and validated by Pedro Pechorro.^23^

### Moral Competence Test (MCT-T)

MCT-T measures moral competence, which were defined by Lind^24^^,^^25^ moral orientations for problem and conflict solving – thinking and discussion on the moral quality of the argument rather than in regard to their opinion-agreement. MCT-T scores range from 0-100 and have shown good validity. While there are many tests of moral preferences or attitudes, the MCT-T is one of the few instruments, if not the only, which produces a pure measure of moral competence, and contains a moral task for the participant. The scale is composed of three moral dilemmas with 13 questions each: the workers, the doctor and the judge dilemmas. For each dilemma, there are three sections: Section A, in which the subject must answer on a scale from -3 ("I strongly disagree") to +3 ("I strongly agree") how much he agrees with the action led by the workers/doctor/judge; Section B, in which the subject must decide, on a scale from -4 ("I strongly disagree") to +4 ("I strongly agree"), how much he agrees with the arguments in favor of the workers/doctor/judge; Section C, in which the subject must decide, on a scale from -4 ("I strongly disagree") to +4 ("I strongly agree"), how much he agrees with the arguments against the workers/doctor/judge actions. The questionnaire has a total of 13 questions for each dilemma. It was translated and validated to Portuguese by Bataglia and colleagues.^26^

### Setting and procedure

The scales (described in "Data Collection Method") were provided in paper format at the end of class (after asking for students' voluntary participation). Filled questionnaires were collected by a previously assigned university worker. One of the authors digitalized the data into the SPSS, and the other author performed a review. The sample was collected between 1 August 2018 and 1 February 2019.

### Statistical analysis

We used descriptive statistics, means and standard deviation for continuous variables and absolute number and frequencies for categorical variables. Non-parametric tests were employed when the assumptions for parametric null hypothesis tests were violated.

**Table 2:** Overall scores and between-group differences for BLRI, TEMPS-A, and the MCT-T

Variable	TOTAL N=138 Mean (std) Median	1st Year N=104 Mean (std) Median	5th Year N=34 Mean (std) Median	t	U	p-value
ICU Unemotional	7.34 (3.39) 7.00	7.25 (3.38) 7.00	7.62 (3.43) 7.5		1876.5	0.590
ICU Uncaring	5.16 (3.54) 5.00	4.88 (3.70) 4.00	6.00 (2.92) 6.00		2154.5	0.055
ICU Callousness	4.93 (2.68) 5.00	4.85 (2.62) 4.00	5.18 (2.90) 5.00		1888.5	0.549
ICU Total Score	17.43 (7.12) 16.00	16.98(7.22) 16.00	18.79 (6.74) 19.00		2030.5	0.194
BL Level of Regard	17.38 (5.53) 18.00	17.31 (5.43) 18.00	17.62 (5.91) 18.50		1878.5	0.584
BL Empathic understanding	11.51 (8.21) 12.00	11.83 (8.48) 12.00	10.56 (7.36) 10.00	0.780		0.437
BL Unconditionality	5.70 (7.39) 6.00	6.30 (6.41) 6.00	3.85 (9.69) 5.50		1516.0	0.213
BL Congruence	5.83 (8.48) 5.00	5.99 (8.37) 5.00	5.32 (8.93) 4.50	0.397		0.692
BL Total	40.42 (22.68) 40.00	41.42(22.48) 43.00	37.35(23.35) 37.00	0.908		0.366
TEMPS-A Depressive	0.41(0.15) 0.38	0.42(0.14) 0.43	0.38(0.17) 0.33	1.134		0.191
TEMPS-A Cyclothymic	0.36(0.23) 0.33	0.37(0.21) 0.33	0.32(0.29) 0.21	0.962		0.341
TEMPS-A Hyperthymic	0.49(0.20) 0.48	0.50(0.20) 0.48	0.45(0.19) 0.43	1.193		0.235
TEMPS-A Irritable	0.25(0.17) 0.24	0.25(0.16) 0.24	0.26(0.20) 0.20		1732.0	0.858
TEMPS-A Anxious	0.44(0.21) 0.46	0.46(0.20) 0.46	0.36(0.21) 0.33	2.359		0.020
MCT-T	19.03(11.56) 17.00 (N=96)*	18.92(11.39) 17.00 (N=62)*	19.24(12.05) 17.00 (N=34)*		1059.0	0.969

Comparison between 1st and 5th year students. Considering the distribution of data, we adopted t-test for independent variables or Mann-Whitney non-parametric test. Mean and standard deviation are reported. P values correspond to differences between the two groups. * Only 96 students fulfilled the MCT-T (62 from 1st year and 34 from the 5th year).

Pearson r was used to analyze the correlation between empathy and callous traits, moral competencies, and specific traits. When not otherwise specified, two-tailed p<0.05 was considered significant. Given the exploratory nature of this small-sample study, we decided not to control for family-wise error rate, which would have decreased statistical power. All analyses were performed using SPSS IBM 24.

## Results

We found no statistical evidence to support any difference in empathy levels, measured by BLRI, between 1st (N=104, Mean: 41.42, SD: 22.48) and 5th-year students (N=34, Mean: 37.35, SD: 23.35), being that t_(__136)_=0.908, p=0.366. Similarly, we found no statistical evidence to support any difference between the two medical years (Mdn(1st year)= 17.00; Mdn(5th year)= 17.00) regarding Moral competencies, as measured by MCT-T (U=1059.0, p=0.969) and callous traits, as measured by ICU-T (Mdn(1st year)=16.00, Mdn(5th year)=19.00, U=2030.5, p=0.194). To note, we found no statistical evidence for differences between the two groups regarding the subscales of BLRI, MCT-T or ICU-T. Regarding Temperament (TEMPS-A), we found no statistical evidence to support any difference between the two groups, except for the anxious temperament (t_(__136)_= 2.359, p=0.020), which was higher on first-year students. See Table 2, which presents overall scores and tests between the 1st and 5th-year students.

We found a negative correlation between empathy, as measured by BLRI, and callous traits, as measured by ICU-T (r_(__136)_=-0.444, p=0.000). Noteworthy, (1) the ICU-T subscales were negatively correlated with BLRI: unemotional (r_(__136)_=-0.306, p=0.000), uncaring (r_(136)_=-0.402, p=0.000), callousness (r_(136)_=-0.261, p=0.002); Regarding empathy and Moral competences, we found no correlation between the two variables (r_(96)_=0.029, p=0.779).

We explored the correlation between empathy and specific temperaments (anxious, irritable, hyperthymic, depressive, and cyclothymic) and the results are presented in Table 3. Among these results, we would like the reader to notice that: (1) Cyclothymic, anxious and irritable temperaments were negatively correlated with BLRI (r_(__136)_=-0.334, p=0.000, r_(136)_=-0.281, p=0.001, r_(136)_=-0.400, p=0.000); (2) There was no correlation between hyperthymic or depressive temperament and BLRI or between MCT-T and BLRI (see Table 3).

**Table 3:** Pearson's correlation between BLRI total score and TEMPS-A, ICU-T and MCT-T

Variable	BLRI Total Score N=138
TEMPS- D	
Pearson Correlation	-0.085
p value	0.319
TEMPS-CYC	
Pearson Correlation	-0.334
p value	0.000
TEMPS- HYP	
Pearson Correlation	0.473
p value	0.062
TEMPS- ANX	
Pearson Correlation	-0.281
p value	0.001
TEMPS- IRRI	
Pearson Correlation	-0.400
p value	0.000
MCT-T (N=96)*	
Pearson Correlation	0.029
p value	0.779
ICU TOTAL	
Pearson Correlation	-0.444
p value	0.000
ICU UNCARING	
Pearson Correlation	-0.402
p value	0.000
ICU UNEMOTIONAL	
Pearson Correlation	-0.306
p value	0.000
ICU CALOUSNESS	
Pearson Correlation	-0.261
p value	0.002

*Only 96 students fulfilled the MCT-T; Abbreviations: D: Depressive, CYC: cyclothymic, HYP: Hyperthymic, ANX: anxious, IRRI: irritable

## Discussion

Relational and moral competencies (RMC) are classically considered relevant skills in medical practice. This is the first empirical investigation of RMC competencies, including callous traits, in a sample of 1st and 5th-year medical students. Our first aim was to determine Lisbon University Medical School student's empathy, moral competencies, callous traits, and temperament scores and identify potential differences in scores between the 1st and 5th-year medical years.

As for Moral competencies, our sample (N=96, Mean(MCT)= 19.03, SD: 11.56) scored more than other youth populations, as for example, a sample of 827 high school students from urban Polish schools (N=827, Mean(MCT)=14.42, SD: 9.99)^27^ and a sample of teens with a criminal record (N=23, Mean(MCT)= 16.97, SD: 6.2).^28^ On the other hand, medical students scored less than doctors (N=236, Mean (MCT)=22.43, SD: 14.1)^29^, which can lead us to think that Moral Competences may evolve with age and maturity.

Regarding callous traits, medical students scored lower on ICU-T total, callousness, and uncaring (N= 138, Mean (ICU-T): 17.43, SD: 7.12; Mean(ICU-callousness): 4.93, SD: 2.68; Mean (ICU-uncaring): 5.16, SD: 3.54) than adolescents from basic and high school (N=1443, Mean (ICU-T): 24.58, SD: 2.78; Mean (ICU-callousness): 7.29, SD:4.39, Mean (ICU-uncaring): 8.14, SD: 3.98).^30^ We speculate that adolescents who aim for a medical curriculum could be more sensitive and have better interpersonal skills, which per se would determine their interest in working closely with people. Yet previous studies of juveniles with a criminal record suggest that our sample scores are lower in respect to callousness and uncaring traits 31 (N=500, Mean (ICU-Callousness)= 13.75, SD: 5.08; Mean (ICU-uncaring): 15.41, SD: 3.05), while similar in unemotional scores (N=500, Mean (ICU-unemotional)= 7.07, SD: 2.31). In our sample, "unemotional" traits might refer to students believing that they should be guarded in their emotions (having idealized the role of "being professional" as avoiding 'personal feelings' or 'emotions') and therefore answer that taking control of one's feelings when exposed to several impacting experiences as dissecting a cadaver, drawing a patient's blood, diagnosing a terminal illness – these are 'emotional' experiences that they must manage 'unemotionally'.

With respect to temperament, the students scored higher on the depressive, hyperthymic, and anxious dimensions (N=138, Mean TEMPS-Depressive=0.41, SD:0.15, Mean TEMPS-Hyperthymic:0.49, SD:0.20, Mean TEMPS-Anxious:0.44, SD:0.21). In fact, some of the students from our sample had a family psychiatric history and/or current psychotropic treatment that could partly explain these results. When comparing our results to the ones by Jaracz, who studied 100 civil workers and 100 nurses from Poland, we found that: (1) our sample had higher scores on depressive temperament than civil workers (N=100, Mean TEMPS-Depressive=0.26 SD:0.12) and nurses (N=100, Mean TEMPS-Depressive=0.28, SD:0.11) and (2) medical students scored higher on anxious temperament than civil workers (N=100, Mean TEMPS-Anxious=0.26, SD:0.22)  and nurses (N=100, Mean TEMPS-Anxious=0.32, SD:0.22).^32^ Notably, when comparing to Hinic's work with 770 healthy university students (medicine, philology, economics, humanities, sports, engineering and natural sciences, musical and visual arts) we also found that our sample scored higher on depressive and anxious temperament (N=770, TEMPS-Depressive=0.15, SD:0.20,  N= 770, TEMPS-Anxious=0.36, SD:0.25).^33^  If we focus on Hinic's subset of Serbian medical students, our sample still scores higher on these specific temperaments (N=100; TEMPS-Depressive=0.16, SD:0.14, Mean TEMPS-Anxious=0.40, SD:0.2).

Regarding empathy development among the Lisbon University medical students, we found no differences between 1st and 5th-year medical students. Previous studies provide assorted evidence of either an empathy decline (key factors: stress and learning overload, disease-centred approach, lack of student-patient time, abusive tutors, and students' low self-images)^34^^-^^39^ or preservation and even increase.^12^^,^^40^ Interestingly, our results are along with previous studies in Portuguese populations  (t_(136)_=0.908, p=0.366), corroborating the results from the Portuguese work by Magalhães and colleagues: "nonsignificant differences on empathy scores (...) were found between the pre-clinical (...) and clinical phases".^40^^,^^41^ Although we cannot exclude our result to be a false negative it could be that Portuguese medical curriculums attend and act towards caring for the factors that would determine empathy erosion.^42^  

Our second aim was to identify whether empathy was correlated with morality (positively) and callosity (negatively) or not. Moral reasoning wasn't related to empathic skills in our sample (no significant correlation between MCT-T and BLRI scores), and, as such, the classical idea that empathy and moral competencies aren't related could be put forward. Although these results could be due to small sample size (false negative, as only 96 students completed the MCT-T questionnaire), some literature provides evidence that empathy and morality are not in fact correlated. As an example, Kiehl's study with incarcerated psychopath offenders concluded that "psychopathy as a whole did not predict the ability to understand what is morally wrong".^43^^,^^44^ Furthermore, Batson and colleagues found that empathy can even lead to immoral behavior.^45^ Regarding callous traits, as we expected, we found a negative correlation between them and empathy. In line with our results, previous literature defines callous traits as disregarding/uncaring about the feeling of others, concern for the consequences of his/her actions solely on his/herself rather than their effects on others, even when they result in substantial harm to others.^46^

Finally, we aimed to identify the correlation between specific temperaments and empathy, as measured by BLRI. As we postulated, our work showed a negative correlation between empathy and cyclothymic, anxious, and irritable temperaments (r_(__136)_=-0.334, p=0.000, r_(136)_=-0.281, p=0.001, r_(136)_=-0.400, p=0.000). These temperaments, by definition, comprise various representative aspects of negative emotionality, such as irritability, anger, and frustration.^47^^,^^48^ Since negative emotionality plays a crucial role on interpersonal relationships, and therefore empathy, it explains our results. On the other hand, contrary to what we were expecting, hyperthymic temperament was not correlated with empathy. According to Akiskal, hyperthymic individuals are "habitually cheerful, sociable, self-assured, boastful, improvident, uninhibited, overtalkative".^47^ These characteristics seems to have a major role on positive affect ("the display of pleasure and excitement, and the tendency to seek out and approach reward-fulfilling stimulation")^47^ which have been described as essential aspects for social interaction, and therefore empathy. Although it is possible that our results are false negatives, these results hint at a nonlinear relationship between empathy, temperament and positive affect. These findings support the need for a multifactorial definition of empathy, comprising not only emotional, but also cognitive and behavioral dimensions.^3^^,^^4^

### Limitations

Firstly, our study should be considered exploratory due to our small sample size, and we cannot exclude our results from being false negatives or positives. The generalization of our results might be limited by the specifics of our sample selected from a single university (Lisbon University Medical School). Secondly, the use of self-reported scales is associated with various limitations: 1) the answers are dependent on the honesty of participants, 2) the use of scales with subjective questions may difficult its understanding/interpretation and the introspective ability to provide an accurate answer, and 3) its use may have skewed our population, as people prone to answering questionnaires, and therefore with different personal characteristics, are more likely to have answered it. Finally, the constructs of empathy and moral competencies were shown to be heterogeneous both at a conceptual and empirical level, limiting the comparison of our results to other samples.

## Conclusions

Our study aimed to determine empathy, moral competencies, callous traits, and temperament scores on Lisbon University Medical School students. We also had the objective of identifying potential differences in scores between the 1st and 5th-year medical years and the correlation between empathy and morality, callosity and, specific temperaments. To the best of our knowledge, this is the first work that studies empathy, callous trains, morality, and temperament as a whole in medical students. It is also the first work that analyzes the relationship between empathy and temperament.

Regarding the overall scores for the studied variables, we would like the reader to notice that medical students scored lower than the general youth population on callous traits (as measured by ICU-T) and higher on moral competencies (as measured by MCT). These results underline the fact that medical students usually have a specific profile which makes them choose a career with close contact to people. However, we found that medical students had similar scores on unemotional traits when compared to juveniles with a criminal record.  We hypothesized students idealized the role of "being professional" as avoiding 'personal feelings' or 'emotions'. In contrast to the majority of studies on empathy in medical students, which postulate a decrease of empathy throughout medical school, we found no statistical significant difference between 1st and 5th-year medical students. Our results are in line with previous Portuguese studies on the matter, suggesting that Portuguese medical school may have specific characteristics or teaching methods that preserve empathy. On the relationship between empathy and anxious, irritable and, cyclothymic temperaments, we found that they are inversely correlated. As such, the characteristics of an individual medical student may have a major role in the way he will empathize with future patients and colleagues. Finally, we found no correlation between empathy and moral competence, corroborating the findings in previous publications, where it was shown that psychopaths have the same ability to distinguish morally wrong and good.

From the results of our work, it is of utmost importance to admit students into medical school based not only on their curriculum and grades but also on individual characteristics, such as temperament and callous traits. Based on these principles, we will be able to train doctors with higher empathy levels, providing better standards of care in the future.

### Acknowledgements

We would like to acknowledge the students that voluntarily participated in our research.

### Conflict of Interest

The authors declare that they have no conflicts of interest.
